# Economic evaluation of emergency obstetric care training: a systematic review

**DOI:** 10.1186/s12884-017-1586-z

**Published:** 2017-12-04

**Authors:** Aduragbemi Banke-Thomas, Megan Wilson-Jones, Barbara Madaj, Nynke van den Broek

**Affiliations:** Centre for Maternal and Newborn Health, Liverpool School of Tropical Medicine, L3 5QA, Liverpool, UK

**Keywords:** Emergency obstetric care, Training, Economic evaluation, Cost analysis, Cost-effectiveness analysis, Cost-utility analysis, Cost-benefit analysis, Value-for-money

## Abstract

**Background:**

Training healthcare providers in Emergency Obstetric Care (EmOC) has been shown to be effective in improving their capacity to provide this critical care package for mothers and babies. However, little is known about the costs and cost-effectiveness of such training. Understanding costs and cost-effectiveness is essential in guaranteeing value-for-money in healthcare spending. This study systematically reviewed the available literature on cost and cost-effectiveness of EmOC trainings.

**Methods:**

Peer-reviewed and grey literature was searched for relevant papers published after 1990. Studies were included if they described an economic evaluation of EmOC training and the training cost data were available. Two reviewers independently searched, screened, and selected studies that met the inclusion criteria, with disagreements resolved by a third reviewer. Quality of studies was assessed using the Consolidated Health Economic Evaluation Reporting Standards statement. For comparability, all costs in local currency were converted to International dollar (I$) equivalents using purchasing power parity conversion factors. The cost per training per participant was calculated. Narrative synthesis was used to summarise the available evidence on cost effectiveness.

**Results:**

Fourteen studies (five full and nine partial economic evaluations) met the inclusion criteria. All five and two of the nine partial economic evaluations were of high quality. The majority of studies (13/14) were from low- and middle-income countries. Training equipment, per diems and resource person allowance were the most expensive components. Cost of training per person per day ranged from I$33 to I$90 when accommodation was required and from I$5 to I$21 when training was facility-based. Cost-effectiveness of training was assessed in 5 studies with differing measures of effectiveness (knowledge, skills, procedure cost and lives saved) making comparison difficult.

**Conclusions:**

Economic evaluations of EmOC training are limited. There is a need to scale-up and standardise processes that capture both cost and effectiveness of training and to agree on suitable economic evaluation models that allow for comparability across settings.

**Trial registration:**

PROSPERO_CRD42016041911.

**Electronic supplementary material:**

The online version of this article (10.1186/s12884-017-1586-z) contains supplementary material, which is available to authorized users.

## Background

Improving maternal and newborn health has been at the forefront of the global health agenda for more than two decades. However, despite a 44% drop in maternal mortality ratio between 2000 and 2015, an estimated 300,000 women still die each year due to complications of pregnancy and childbirth [1]. In addition, an estimated 2.6 million babies are stillborn and 2.7 million newborns die within the first 28 days of life [2, 3]. Unlike many other public health concerns, maternal and newborn mortality is significantly influenced by institutionally-based clinical interventions [4, 5]. Evidence suggests that majority of these deaths could be prevented by timely and effective emergency obstetric care (EmOC) [6, 7]. However, recent evidence shows that more than half of all women with obstetric complications lack access to this life-saving intervention [8]. EmOC relies on the presence of suitably trained and competent healthcare providers. When carried out by a competent provider, it is estimated that EmOC can reduce intra-partum stillbirths by between 45% and 75%, [9] as well as reduce health facility-based maternal mortality by up to 50% [10].

In the early 1990s, there was wide acknowledgement globally that deficiency in the obstetric skills of healthcare providers was one of the reason for poor quality of care. In view of this, training for healthcare providers was recommended [11, 12]. At the time, EmOC training courses such as the Advanced Life Support in Obstetrics (ALSO) and Managing Obstetric Emergencies and Trauma (MOET) were developed to meet this need in high income settings [13, 14]. Since then, several other training programmes have been developed and implemented across the globe [15–18]. Studies have shown that in-service EmOC training is effective in increasing knowledge and skills of healthcare providers and can improve the quality and effectiveness of care [19–23]. However, despite this, very little is published about the costs and cost-effectiveness of training. Such information is usually obtained via economic evaluation studies. Partial economic evaluations (such as cost analysis, cost-description or outcome description), consider costs and/or consequences but do not compare different interventions or do not relate costs to benefits. Full Economic Evaluations (such as cost minimisation analysis (CMA), cost-effectiveness analysis (CEA), cost-utility analysis (CUA) and cost-benefit analysis (CBA)), compare both the costs and the consequences (benefits, effectiveness) of one or more interventions [24] (Table 1).

**Table 1:** Description of types of economic evaluation studies

Type of economic evaluation	Description
Partial economic evaluation	▪ Cost analysis: Compares the costs of alternative interventions.
	▪ Cost of illness study: Identifies and measures the total costs attributable to a specific disease.
	▪ Cost description: Examines the costs of a single intervention or programme (which can have multiple interventions).
	▪ Outcome description: Examines only the consequences of a single intervention or programme.
Full economic evaluation	▪ Cost-minimization analysis (CMA): Comparison of costs (monetised) when there is proven evidence of equivalent effectiveness of the interventions or programs being compared.
	▪ Cost-effectiveness analysis (CEA): Cost is monetised while effectiveness is measured in “natural units” such as life-years gained, lives saved.
	▪ Cost-utility analysis (CUA): Cost is monetized while ‘effectiveness’ is measured as a utility such as Quality-adjusted life years (QALYs) or Disability-adjusted life years (DALYs). Both QALYs and DALYs are composite metrics of length and quality of life.
	▪ Cost benefit analysis (CBA): Costs and benefits are both monetised.

In the era of the Sustainable Development Goals (SDGs), when competition for limited resources is high, information on the cost-effectiveness of existing and promising new interventions to improve health of mothers and their babies will be central to informing policy and practice [25]. It is important to understand the costs and cost-effectiveness of training packages in order to aid decision-makers on the most efficient use of resources and to assess value-for-money [25, 26]. The objective of this review is to systematically assess and summarise the evidence available on economic evaluations of in-service training in Emergency Obstetric Care (EmOC) for healthcare providers.

## Methods

In designing the methods for this review, we borrowed critical insights on best practices for conducting systematic reviews of economic evaluations from experts from the Centre for Reviews and Dissemination and the Task Force on Community Preventive Services [27, 28].

The Preferred Reporting Items for Systematic Reviews and Meta-Analyses (PRISMA) approach was used in reporting the findings of the systematic search conducted for this review [29].

### Search strategy

Multiple strategies were used to search articles in PubMed, Scopus, the Cochrane Library, Web of Knowledge, Google Scholar, CINAHL Plus, Global Health Archive, EconLit, Popline and African Journal Online. In searching, we combined medical subject headings (MeSH) and/or key words, using Boolean linkages “OR” within categories and “AND” between three categories.

- Emergency obstetric care: “obstetric emergenc*” OR “emergency obstetric care” OR “emergency obstetric and newborn care” OR EmOC OR EmONC
  AND
- Training: Training OR education OR “capacity building”
  AND
- Costs and economic evaluation: “cost*minimization” OR “cost*analysis” OR cost* OR “cost*effectiveness” OR “cost*utility” OR “cost*benefit” OR “economic evaluation”

The search terms used were based on the optimal search strategy for retrieving cost and economic studies in health services research [30].

The websites of non-government organisations and UN agencies were searched to identify grey literature, including John Snow International (JSI), Population Council, Averting Maternal Death and Disability (AMDD), Maternal Health Task Force (MHTF), United Nations Children’s’ Fund (UNICEF), United Nations Fund for Population (UNFPA) and World Health Organization (WHO). In addition to the automated search, relevant articles were identified through searching reference lists by hand and reviewing studies included in systematic reviews of training effectiveness.

The search was conducted for articles published from January 1990 to December 2016. The decision to include only studies published from 1990 was based on the recognition that in-service EmOC training was introduced at this time. We did not limit our search by language.

Two co-authors independently conducted the search and screened all retrieved records. Titles and abstracts were screened for relevance and eligibility, based on the set inclusion and exclusion criteria. Any discrepancies were resolved through discussion with the other co-authors. This was done to minimise selection bias.

### Inclusion and exclusion criteria

Articles were included if these described any type of economic evaluation of an in-service (as opposed to pre-service) training in EmOC and provided results of the evaluation including costs data.

Articles (including letters, commentaries or editorials) that reported effectiveness data without any training cost data were excluded. In addition, articles that reported multiple implemented trainings without disentangling EmOC training specifically and any articles that described training of non-healthcare provider participants were also excluded.

### Data extraction

Information was extracted pertaining to; study and training characteristics (including year of publication, country of training, cadre of training participants, number of training participants, training content, trainers/facilitators, duration of the training) as well as key findings on costs and cost-effectiveness of the training (economic evaluation type, overall study design (standalone evaluation versus nested in another study), the full breakdown of costs included for analysis by authors, reported or estimated total training implementation costs and currency in which costs were reported). Data was extracted by two reviewers independently and then checked for accuracy by a third reviewer.

### Quality assessment

The 24-item Consolidated Health Economic Evaluation Reporting Standards (CHEERS) checklist was used to assess the quality of reporting of the included full economic evaluations [31]. For partial economic evaluations, the relevant criteria in the CHEERS checklist were combined with those suggested by Stone et al. 2005 [32] (including provision of full costs breakdown and inclusion of opportunity costs) to create an eight-item costs focused quality checklist. The opportunity cost was taken to be the value of the best foregone alternative use of resources [24].

For each item, a score of 1 was awarded if the criterion was fully met, 0.5, if partially met, 0, if not met or only minimal information was provided and NA if not applicable. The total score achieved across all the criteria was then summed and converted to percentages. Since no standardised interpretation of the quality assessment tool exists, studies with 75% or more criteria fully met were graded as high quality, 50–74% as average quality and below 50% as poor quality [33]. Each included study was assessed independently by two co-authors.

### Data synthesis

Following a brief description of the characteristics of the type of EmOC training reported, studies were classified as being either a partial or full economic evaluation.

For costs captured in both partial and full economic evaluations, all the cost data provided by the authors were retrieved. The different direct training costs (core costs including costs for central management, monitoring and research and dissemination of findings, implementation costs, overheads and external evaluation) were identified. For cost comparison across the included studies, only implementation costs for the training (costs incurred for actual delivery of the training) were selected and included. Examples of direct implementation costs included in the comparative analysis include cost of hiring a training venue, teaching materials, equipment costs, supervision costs, travel expenses, and/or, consultant fees for trainers. Opportunity costs such as costs of work that trainees could have being doing if they were not attending the training were excluded. Costs associated with start-up (such as cost of setting up an office for the training organisation), administration and capital projects were also excluded.

To allow for cost comparability, 2015 purchasing power parities conversion factors [34] were used to convert local currency of the country in which the training was conducted to International Dollar (I$) equivalents for the year the training took place [35]. Costs reported in US dollars using ‘market exchange rates’ were converted to local currency for the year the training was conducted, using official OANDA exchange rates (http://www.oanda.com/currency/historical-rates). The derived local currency value was subsequently converted to I$ equivalents for the same year. Purchasing power parity (as opposed to market exchange rates) allows one to estimate the amount it would have cost hypothetically to purchase the same market basket of goods in both countries, if their currencies were at par [35]. Based on the I$ equivalents, the cost per trainee per day were calculated for each study. When training was implemented over multiple years, we selected the last year of implementation when the training was completed.

For cost-effectiveness (which is only captured in full economic evaluations), the dimensions used to report effectiveness/utility/benefits in the included studies were identified. A narrative synthesis was used to summarise the available evidence [27].

## Results

**Fig. 1:**
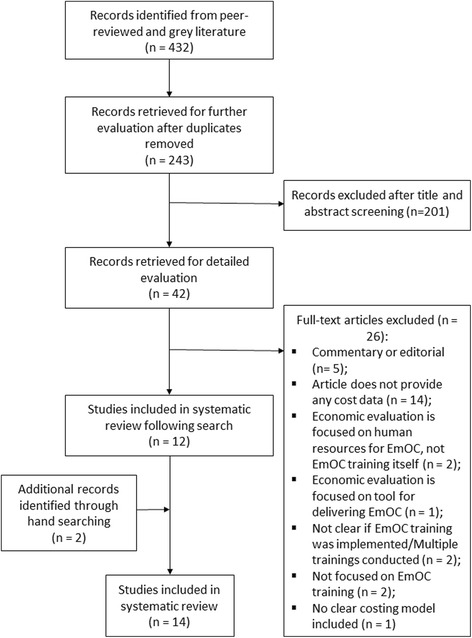
PRISMA diagram

Two hundred thirty nine articles from both peer-reviewed and grey literature sources were screened by title and abstract for inclusion in the full-text review. Full-text of 42 articles were subsequently read, of which 12 articles met the inclusion criteria. An additional two articles were identified following hand searching, with a total of 14 studies included in the analysis (Fig. 1). Details of data extracted from the included studies and reasons for excluding the excluded studies are presented in the summary table (Additional file 1: Table S1: Summary of included studies).

### Overview of studies

Nine studies were partial economic evaluations [36–44] and five studies were full economic evaluations [45–49] (four used CEA [45–48] and one used a mix of CEA and CUA [49]). No CBA study was retrieved. Ten studies were stand-alone economic evaluations [36, 37, 39–45], while four were reported as part of quasi-experimental studies of training effectiveness [38, 46, 47, 49]. All the articles retrieved were published in English.

Eight studies reported on EmOC training alone [37, 42–46, 48, 49], while the remaining six reported on EmOC training conducted along with other interventions such as healthcare facility renovation, improved availability of equipment and supplies, management information systems and reviewed policy or advocacy [36, 38–41, 47].

A total of 11 studies focused on the economic evaluation of a specific EmOC training intervention [36, 44, 47, 49], two studies compared cost-effectiveness of alternative EmOC training delivery approaches [45, 46], and one study compared the cost of training doctors and surgical technicians per surgery conducted after training [48].

The included studies reported on trainings conducted in eleven low- and middle-income countries including Bangladesh [36, 37], Ethiopia [41], Ghana [46], Indonesia [45], Kenya [47], Mozambique [38, 48], Nepal [39], Nigeria [40, 43], Tanzania [47], Zambia [49] and Zimbabwe [42]. One study was conducted in the United Kingdom (UK) [44].

### Quality assessment

Based on the CHEERS checklist [31], one full economic evaluation was rated high quality [48], while the remaining four studies were rated average quality [45–47, 49]. Applying the costs quality criteria to all studies, two partial economic evaluation [39, 44] and all five full economic evaluations [45, 49] were assessed as high quality. Six partial economic evaluations were assessed as average quality [36–38, 40, 41, 43], and one partial economic evaluation was assessed as low quality [43] (Additional file 2: Table S2: Quality assessment of full economic evaluations, Additional file 3: Table S3. Quality assessment of cost analysis in partial and full economic evaluations).

### Description of EmOC training for which economic evaluations have been conducted

Three studies reported on training conducted for midwives only [40, 45, 49], one for doctors only [36], eight for both doctors and midwives [37–39, 41, 42, 44, 46, 47], one for health aides and midwives [43], and one for surgical technicians, who provide EmOC [48].

Most studies described training that lasted between 1 and 30 days (seven studies) [38, 42–46, 49], three medium-term ranging from 45 to 180 days [37, 41, 46], and three described long-term training that ranged from one to three years [36, 37, 48]. Two studies did not specify the duration of the training [40, 47].

In the two studies that provided information on number of trainers and trainees, trainee/trainer ratio ranged from 3:1 to 7:1 [42, 49].

Seven of the trainings reported were facility-based [37, 38, 40–42, 44, 49], one was fully residential [39], while another four were of a mixed format (facility based with residential or facility-based with self-paced learning) [43, 45, 46, 48]. Two studies did not define the training site [36, 47].

Number of healthcare providers trained in the included studies ranged from 10 midwives in Nigeria [40] to 477 providers including doctors and midwives in the UK [44]. The number of trainees per session ranged from 18 to 28 [42, 49].

### Costs of implementing EmOC training

The most commonly included costs for EmOC training were training materials (11 studies) [37–39, 41–46, 48], and travel expense and subsistence fees for facilitators (10 studies) [36–38, 41, 43–49].

The least commonly included costs were participant’s catering and boarding (three studies each) [43, 47, 49], and administration costs [9, 42, 47]. Two studies estimated the opportunity cost of the time spent by healthcare providers attending the training (instead of providing health care) [44, 46].

Three studies [40, 41, 46] included the actual component cost and/or percentage breakdown of the total training implementation costs. In Nigeria, the training equipment (52%) and obstetrician visits (30%) were the mostly expensive components of the training implementation cost [40]. Similarly, in Ethiopia, 67% of the total implementation cost was spent on training materials and equipment while the remaining 33% was spent on travel expenses and per diems [41]. However, in the training conducted in Ghana, per diem constituted the largest proportion of training implementation costs, making up 63% (self-paced learning) and 75% (residential) respectively. This was followed by resource person allowances (18% (self-paced learning) and 17% (residential)) [46].

It was possible to estimate the cost per trainee per day for nine of the included studies [36–38, 43–46, 48, 49] (Table 2). This ranged from I$5 to I$90 per trainee per day [36, 45]. Trainings that required boarding cost between I$33 and I$90, while those that were facility-based or, had some form of internship incorporated, cost between I$5 and I$21 per trainee per day. It was not possible to estimate costs per trainee per day for the other studies because the number of training days [40, 47] or the training implementation costs [39, 41, 42] were not provided.

**Table 2:** Cost of Emergency Obstetric Care training for included studies

S/No	Author(s)	Country of training	Number of trainees	Year training was conducted	Duration of training (Days)	Implementation costs reported or estimated (US$)	Local country currency	Implementation costs (Local currency)	Implementation costs (I$)	US$ to local currency conversion rate	Purchasing Power Parity (PPP) conversion factor	Cost/trainee/day (I$/trainee/ day)
1	Oyesola et al., 1997	Nigeria	10	1992	–	2090	Naira	45,750	1245	22	36.76	–
2	Chukudebelu et al., 1997	Nigeria	64	1992	21	13,000	Naira	284,570	7741	22	36.76	6
3	Walker et al., 2002	Indonesia	110	1998	14	35,171	Rupiah	355,479,628	221,179	10,107	2576.11	90
			284	1998	11	71,722	Rupiah	724,907,164	427,552	10,107	2576.11	90
			48	1998	30	2777	Rupiah	28,067,639	66,945	10,107	2576.11	8
4	Mekbib et al., 2003	Ethiopia	27	1999	45	48175^b^	Tanzanian Shillings	34,926,875	13,970,750	725	2.50	–
			7	1999	90	(included above)	Tanzanian Shillings	(included above)	(included above)	725	2.50	–
5	Gill & Ahmed, 2004	Bangladesh	1	1999	365	900	Taka	44,010	1930	48.9	22.80	5
6	Osei et al., 2005	Ghana	75	2002	21	51,463	Cedisa	51,463	51,463	1.00	1.00	33
			40	2002	180	79,327	Cedis^a^	79,327	79,327	1.00	1.00	11
7	Islam et al., 2006	Bangladesh	14	2004	365	21,700	Taka	1,251,439	50,830	57.67	24.62	10
			21	2004	119	21,420	Taka	1,235,291	50,174	57.67	24.62	20
8	Santos et al., 2006	Mozambique	137	2003	28	144,083	New Metical	3,338,403	326,654	23.17	10.22	85
9	Rana et al., 2007	Nepal	19	2004	42	–	Nepali Rupee	–	–			–
			2	2004	119	–	Nepali Rupee	–	–			–
10	Boulenger & Dmytraczenko^c^, 2007	Kenya and Tanzania	167	2006	–	305,015	Shillings	–	–			–
11	Kruk et al., 2007	Mozambique	53	1996	1080	776,132	New Metical	7,963,114	1,212,042	10.26	6.57	21
12	Manasyan et al., 2011	Zambia	18	2005	5	2880	Kwacha	12,690,374	4951	4406.38	2563.23	18
13	Crofts et al., 2015	Zimbabwe	27	2011	1	6000	Zimbabwean Dollars	2,238,000	1376	373	1625.91	–
14	Yau et al., 2016	United Kingdom	477	2016	1	42,829	British Pounds	30,816	21,263	0.898313	0.69	45

^a^ Cedis no longer used in Ghana, amount stated in US dollars

^b^ Costs for specific EmOC training not isolated

^c^ Duration of training not reported

### Cost-effectiveness of EmOC training

One study used knowledge change of the group (mean score) for labour and delivery [46] and another used change in mean skills score [45]. Four studies reported effectiveness as the mean number of deliveries attended [45], performance change for managing obstetric and other complications [46], change in proportion of deliveries conducted by a skilled birth attendant [47] and change in number of major obstetric surgeries conducted [48]. One study used both number of lives saved and number of Disability Adjusted Life Years (DALYs) [49] (Table 3).

**Table 3:** Cost-effectiveness of training in economic obstetric care (EmOC)

Author(s)	Economic evaluation type	Perspective	Effectiveness metric utilised	Effectiveness	Cost-effectiveness reported	Value for money statement	Sensitivity analysis
Walker et al., 2002	Cost-Effectiveness Analysis	Healthcare provider	Change in scores for skills Cost per additional skilled midwife	All programmes resulted in statistically significant improvements (*P* = 0.03) in the skills of healthcare providers.	Advanced LSS: US$49.7 per 1% increase in mean skill scores and US$3210.9 per % point increase in the number of competent facility midwives.Basic LSS: US$60.7 per % point increase in mean skill scores and US$5651.5 per % point increase in the numbers of competent village midwives.Village midwives internship: US$154.0 per % point increase in mean skill scores and US$4060.8 per % point increase in the number of competent village midwives.	Not clear whether the training programmes were more or less cost-effective than other safe motherhood interventions because the nature of the outcome measures hindered comparison.	Done
Osei et al., 2005	Cost-Effectiveness Analysis	Not defined	Knowledge change of provider on how to conduct labour and delivery,Performance with regard to managing obstetric and other complications	Knowledge changeSelf-paced Learning (SPL): 17% change from baseline to endlineResidential (R): −5%Performance changeSPL: 6% performance change from baseline to endlineR: 4% performance change from baseline to endline	Knowledge changeSPL: US$69 per provider per % point change R: Not calculated due to the negative change in the indicator from baseline to endline.Performance changeSPL: US$101 per provider per % point changeR: US$138 per provider per % point change	Not reported	Not done
Boulenger & Dmytraczenko, 2007	Cost-Effectiveness Analysis	Government	Cost of skilled care per delivery	–	The average annual cost of the skilled care per delivery with a skilled birth attendant was US$15.0 for Tanzania, and US$10.6 for Kenya. The cost per capita was US$1.7 for Tanzania, and US$0.6 for Kenya.	Not possible to compare to similar interventions.	Not done
Kruk et al., 2007	Cost-Effectiveness Analysis	Modified societal perspective	Cost of surgeries conducted	–	The resulting cost per surgery for surgical technicians is US$38.87 versus US$144.1 for physicians.	Surgical technicians retained a substantial cost advantage in all the scenarios.	Done
Manasyan et al., 2011	Cost-Effectiveness Analysis, Cost-Utility Analysis	Not defined	Number of lives saved	97 lives saved. All-cause 7-day neonatal mortality decreased from 11.5 per 1000 to 6.8 per 1000 after training (relative risk: 0.59 (0.48–0.77); *P* < .001) and was associated with a decrease in deaths caused by birth asphyxia (3.4–1.9 per 1000; *P* = .02) and infection (2.1–1.0 per 1000; *P* = .02)	The intervention costs were US$208 per life saved and US$5.24 per disability-adjusted life-year averted.	Considered value for money as Gross Domestic Product (GDP) per person in Zambia was about $1500.	Not done

Walker et al. reported cost-effectiveness/utility as cost per 1% increase in mean skills scores, by comparing the cost-effectiveness across three in-service training programmes in Indonesia [45]. Osei et al. comparing the cost-effectiveness of a traditional residential and a self-paced learning approach, used cost per unit improvement in participant knowledge and skills [46]. The authors noted that the most cost-effective approach to training was dependant on the specific knowledge or skill being taught, and whether opportunity costs were included along with implementation costs in the cost analyses. Osei et al. measured opportunity costs as the value of personnel time both in terms of trainers and participants. Although the self-paced learning approach cost more than the residential approach, this was considered to be more cost-effective than the residential approach with regard to improving knowledge, when direct implementation costs alone were considered (i.e. excluding opportunity cost). When both opportunity and implementation costs were considered, the residential approach proved more cost-effective [46].

Manasyan et al. reported the cost per DALY [49]. In this cost utility analysis, the cost per DALY averted was calculated from the cost per life saved (cost of training divided by the reduction in mortality) divided by the life expectancy in Zambia at the time of study. The authors estimated a ratio of US$5.24 per DALY averted, which when compared with a GDP per person in Zambia of about US$1500 (WHO cost-effectiveness threshold for the country [50]), suggests that the EmOC training intervention could be considered to be good value-for-money [49].

## Discussion

### Main findings

Overall, 14 studies were identified which conducted an economic evaluation of healthcare provider training in Emergency Obstetric Care. Of these, five were full and nine were partial economic evaluations. Training equipment, per diems and resource person allowances or facilitator fees were the most expensive cost components. When cost estimates were inflated to a constant price year, it cost between I$5 and I$90 to train a participant per day. Training that require participants to stay in accommodation (hotel or other) away from their place of work cost more compared to training which is health facility-based (Range I$33-I$90 vs I$5-I$21, respectively). Comparable effectiveness metrics such as Disability and Quality Adjusted Life Years were rarely used. The methods used in estimating cost-effectiveness varied considerably amongst studies.

### Strengths and limitations

To the best of our knowledge, this is the first systematic review of economic evaluations of in-service training in Emergency Obstetric Care. We included both peer-reviewed and grey literature. Meaningful comparisons were possible with regards to costs per trainee per day, using the purchasing power parity equivalents of the training implementation costs. However, it is not possible to do the same for cost-effectiveness, because of the very different measures of effectiveness used in the included studies which ranged from immediate change in knowledge and skills of healthcare providers to the estimated number of lives saved.

### Interpretation

Although many implementation programs include training in Emergency Obstetric Care [51–53], comparatively few studies have reported costs and/or cost-effectiveness of such trainings. This review illustrates that studies of average or low quality were those conducted as part of other studies. Similar observations were made in a systematic review [33]. The three main reasons for a low quality scores were a failure to; 1) provide a detailed breakdown of implementation costs, 2) include indirect and intangible costs (such as loss of productivity), or 3) describe the perspective of the economic study (government, society or healthcare provider). This information is critical for interpretation of economic evaluations and researchers should be encouraged to capture these details in future to improve the quality of published economic evaluation studies.

The way in which costs are measured and valued can have a substantial impact on the overall cost of an intervention and, therefore, the cost-effectiveness. In all the partial economic evaluations conducted, the costs of implementing the training were calculated using the financial definition of costs (the actual expenditure). Only three studies included ‘opportunity costs’ [44, 46, 48], which allows for more comprehensive economic analyses. Although this leads to higher overall costs, studies that fail to include opportunity costs are likely to report significantly favourable (rather than actual) cost-effectiveness [54]. With regard to in-service training of healthcare providers, this is particularly important. The opportunity costs associated with healthcare providers spending time away from providing clinical services, can be significant [55]. There is value in estimating both financial and economic costs since the former is the basis for budgeting and the latter is useful for robust full economic evaluations [56]. Finally, transparency regarding all of the costs will help researchers and policy makers to better identify areas where savings can be potentially made to reduce the overall cost of training can be reduced, thereby increasing the value-for-money.

### Implications for practice

For the studies included in the review with relevant information available, it is clear that training equipment, per diems and resource person allowance account for the majority of the costs [40, 41, 46]. Possible cost saving strategies include sourcing training equipment in bulk centrally, establishing multi-purpose skills training rooms or laboratories which can be used to train multiple groups of healthcare providers [17, 57]. Introduction of a “no per diem” policy and paying only for subsistence costs would significantly reduce the cost of training in many settings [42]. There is a need to eradicate a culture where training has become “an opportunity to supplement income”, rather than an opportunity to build professional capacity [42]. Using volunteers, who have the requisite expertise to provide training and ensuring on-the-job training and continuous clinical education is an inherent part of senior staff roles and responsibilities, will save on ‘consultant’ fees [17].

Training which requires boarding of participants (residential or “hotel-based” training) costs significantly more compared to facility-based training. A recent systematic review on impact of Emergency Obstetric Care training showed that there is gathering interest in, and preference for, ‘facility-based’ or ‘on-site’ or ‘in-house’ training [53]. In addition to reducing the cost per trainee per day, this approach increases the potential for scale-up and is more likely to be sustainable.

The use of standard effectiveness measures allows for comparison of cost-effectiveness to be made across interventions. Although effectiveness measures of patient outcomes, are considered as the ‘real’ benefit of providing Emergency Obstetric Care training. [53, 58]. It is methodologically challenging to measure this [59, 60]. Similarly, especially where training is only one component of a larger implementation program, attribution is problematic [61, 62]. It may be methodologically correct to measure the primary outcome to the healthcare provider (knowledge, skills, competency) more systematically across settings for different types of training.

## Conclusion

In-service training in Emergency Obsteric Care is considered to be an effective way to improve knowledge and skills of healthcare providers, which should improve performance, lead to better recognition and management of women who have complications during and after pregnancy or at the time of birth and can potentially reduce morbidity and mortality. In this respect, the wider health, social and economic benefits resulting from relatively small investments in training can be substantial, suggesting that these investments are likely to be good value-for-money [63]. The findings from this review underscore the need for more cost-effectiveness studies while strategically exploring approaches that maximise cost-savings for implementation.

## Additional files

Additional file 1: Table S1. Summary of included studies (DOCX 23 kb)

Additional file 2: Table S2. Quality assurance of full economic evaluations (DOCX 19 kb)

Additional file 3: Table S3. Quality assessment of cost analysis in partial and full economic evaluations (DOCX 15 kb)

## Abbreviations

ALSO: Advanced Life Support in Obstetrics
AMDD: Averting Maternal Death and Disability
CBA: Cost-Benefit analysis
CEA: Cost-Effectiveness Analysis
CHEERS: Consolidated Health Economic Evaluation Reporting Standards
CMA: Cost Minimisation Analysis
CUA: Cost-Utility Analysis
DALYs: Disability Adjusted Life Years
JSI: John Snow International
MHTF: Maternal Health Task Force
MNH: Maternal and Newborn Health
MOET: Managing Obstetric Emergencies and Trauma
SDGs: Sustainable Development Goals
UNFPA: United Nations Fund for Population
UNICEF: United Nations Children’s’ Fund
WHO: World Health Organization
