# Supplementary material for: Economic evaluation of emergency obstetric care training: a systematic review
Source: BMC Pregnancy Childbirth. 2017 Dec 4;17:403. doi: 10.1186/s12884-017-1586-z (PMC5716021; doi:10.1186/s12884-017-1586-z)
Supplement: Supplementary file 1 — Summary of included studies (DOCX 23 kb) [file 12884_2017_1586_MOESM1_ESM.docx]

**Supplementary Table 1: Summary of included summaries**

| **S/No** | **Author(s), Year**  **Country of Training** | **Cadre of training participants and number trained** | **Trainers/ Facilitators** | **Duration of the training** | **Training site** | **Full vs. partial economic evaluation, economic evaluation type** | **Costs included for analysis by authors** | **Training of instructors** | **Training venue** | **Training materials** | **Equipment** | **Supervision/ Mentoring** | **Travel, expense, consultant fees for trainers** | **Travel and subsistence fees for trainees** | **Catering** | **Accommodation** | **Administration** | **Start-up cost** | **Capital projects** | **Opportunity cost** | **Implementation costs reported or estimated** | **Opportunity costs captured** | **Key outcome(s)** | **More on outcomes** | **Cost-effectiveness ratio reported** |
| --- | --- | --- | --- | --- | --- | --- | --- | --- | --- | --- | --- | --- | --- | --- | --- | --- | --- | --- | --- | --- | --- | --- | --- | --- | --- |
| 1 | Oyesola et al, 1997  Nigeria | Midwives  10 Midwives from three facilities, including three from Birnin Kebbi | Physicians, obstetricians, social scientists and midwives hired for this purpose | Not specified | Facility based | Partial economic evaluation, Cost analysis | No training cost details provided | -- | -- | -- | -- | -- | -- | -- | -- | -- | -- | -- | -- | -- | US$2090 | -- | NA | NA | NA |
| 2 | Chukudebelu et al, 1997  Nigeria | Health aides and midwives  36 aides (15 from the private sector, 21 from the public sector) and 28 midwives all from the public sector attended | Not defined | 3 weeks | Facility based but participants travelled to the facility for training | Partial economic evaluation, Cost analysis | Cost of both theoretical and practical training, including design, curriculum development, per diems for trainees’ accommodation, meals and travel and lecture fees paid to resource persons. The | No | Yes | Yes | -- | No | Yes | Yes | Yes | Yes | No | No | No | No | US$13,000 | No | NA | NA | NA |
| 3 | Walker et al, 2002  Indonesia | Midwives  110 (Facility-based format) 284 (Village based format 1) 48 (Village based format 2)  Facility-based midwives in the province of South Kalimantan (Advanced LSS).  Village-based midwives in the province of South Kalimantan (Basic LSS).  Village-based midwives in the province of South Kalimantan (Village midwives internship) | Facility-based midwives | 14 days (Advanced LSS)  11 days (Basic LSS)  30 days (Village midwives internship) | Advanced LSS  Basic LSS  Village midwives internship | Full economic evaluation, CEA | Central administration, tehnical assistance, start-up (training needs assessment, site preparation, training of trainers), operational (life-saving skills, fund-raising, peer review, continuing education) | Yes | Yes | Yes | Yes | Yes | No | No | No | No | Yes | Yes | No | No | Advanced LSS: US$56,374  Basic LSS: US$108,974  Village  midwives internship: US$17,063 | No | Change in mean number of deliveries | All programmes resulted in statistically significant improvements (P 4 0.03) in the skills of FMWs and VMWs | Advanced LSS: US$ 49.7 per 1% increase in mean skill scores and US$ 3210.9 per 1% increase in the number of competent FMWs. Basic LSS: US$ 60.7 per 1% increase in mean skill scores and US$ 5651.5 per 1% increase in the numbers of competent VMWs. Village midwives internship: US$ 154.0 per 1% increase in mean skill scores and US$ 4060.8 per 1% increase in the number of competent VMWs. |
| 4 | Mekbib et al, 2003  Ethopia | General practitioners, midwives and other EmOC service providers  7 general practitioners, 4 midwives, 5 health officers and 18 health assistants from Ambo hospital and the two health centres | A master trainer, trained general practitioner and an obstetrician-gynaecologist | 3 months for General practitioner, 1.5 months for midwives | Facility based | Partial economic evaluation, Cost analysis | Training and seminars, per diem and travel | No | No | Yes | Yes | No | Yes | Yes | No | No | No | No | No | No | US$48,175 | No | NA | NA | NA |
| 5 | Gill & Ahmed, 2004  Bangladesh | Medical officers and family welfare visitors (FWVs)  Number not specified | Not defined | 1 year for medical officers and 6 months for FWVs. | Not defined | Partial economic evaluation, Cost analysis |  | -- | No | -- | Yes | No | Yes | Yes | No | No | No | No | Yes | No | Not stated | No | NA | NA | NA |
| 6 | Osei et al, 2005  Ghana | Midwives and back/up referral physicians  75 (Residential format) 40 (Self-paced learning)  For residential (R) format, trainees came from Jirapa-Lambussie and Mamprusi respectively. For Self-paced learning (SPL) format, Nadowli in Upper West Region and Yendi in Northern Region. | Regional Resource Teams, previously trained by IntraHealth International | 21 days (R format) 180 - 270 days (Self-paced learning format) | Residential and Facility based (Self-paced learning format) | Full economic evaluation, CEA | Travel and transport, per diem, Resource person allowance, other direct costs (Rentals, training supplies etc.) | Yes | Yes | Yes | Yes | No | Yes | Yes | No | No | No | No | No | Yes | Self-pace learning: GHC411,705,615 Residential: GHC634,617,923 | Yes SPL: 6,933,299 R: 1,575,801 | Knowledge change of group mean score of provider on how to conduct labour and delivery, Performance change at managing obstetric and other complication | Knowledge change SPL: 17% change from baseline to endline R: -5% Performance change SPL: 6% performance change from baseline to endline R: 4% performance change from baseline to endline | Knowledge change SPL: $69 per provider per % change  R: Not calculated due to the negative change in the indicator from baseline to endline. Performance change SPL: $101 per provider per % change R: $138 per provider per % change |
| 7 | Islam et al, 2006  Bangladesh | Medical officers and nurses  14 medical officers and 21 nurses | Not defined | 119 days | Facility based | Partial economic evaluation, Cost analysis | Trainers’ honorarium, trainees’ travel and daily allowances, book grant, training materials, expenses related to facility set-up, monitoring and on-site mentor- ing | No | No | Yes | No | Yes | Yes | Yes | No | No | No | Yes | No | No | Not stated | No | NA | NA | NA |
| 8 | Santos et al, 2006  Mozambique | Physicians, surgical technicians, medical and MCH technicians, mid-level nurses, basic nurses and elementary midwives.  137 professionals (132 for BEmOC and 5 for CEmOC) (11 physicians, 4 surgical technicians, 15 medical and MCH technicians, 16 mid-level nurses, 63 basic nurses and 28 elementary midwives) | Not defined | 28 days for basic EOC and 84 days for comprehensive EmONC training. | Facility based | Partial economic evaluation, Cost analysis | Technical training, travel | -- | -- | Yes | Yes | No | Yes | Yes | No | No | -- | No | No | No | US$144,083 | No | NA | NA | NA |
| 9 | Rana et al, 2007  Nepal | Various cadres of health professionals, including specialist general practitioners, non-specialist doctors and nurses.  19 (Competency-based basic EmOC), 2 (Competency-based comprehensive EmOC)  Other training participants included trainers, senior auxiliary health worker, health assistants, electricians, ward-in-charge | Local trainers developed through training-of-trainers curriculum by the John Hopkins Program for International Education in Gynaecology and Obstetrics (JHPIEGO) and AMDD. | 42 days (Competency-based basic EmOC), 119 days (Competency-based comprehensive EmOC) | National training site | Partial economic evaluation, Cost analysis | Needs assessment, Construction, Equipment and supplies, Technical training, Management training, Planning, monitoring, supervision for quality of care, Policy advocacy, Community activities, IEC, advocacy, Project support.  However, does not specifically isolate costs of EmONC training. | -- | Yes | Yes | Yes | Yes | -- | -- | -- | No | No | Yes | Yes | -- | ***Technical training: US$205,660 (Inclusive of all 11 different trainings. Does not isolate EmONC) | -- | NA | NA | NA |
| 10 | Boulenger & Dmytraczenko, 2007  Kenya and Tanzania | Maternity care providers, mainly in primary level health facilities (dispensaries and health centres).  115 (Igunza, Tanzania) 53 (Homabay, Kenya),  49 (Migori, Kenya) | Not defined | Not defined | Not defined | Full economic evaluation, CEA | Consultant fees, facilitator fees, purchase of equipment (water supply, solar equipment, maternal health equipment, communication equipment, etc.), equipment installation, printing costs, costs of meetings and trainings (which include per diems, hall hire, lunches and meals, accommodation, secretarial services, stationeries, supplies and printing), and any travel costs associated with the activities. | Yes | Yes | No | Yes | No | Yes | Yes | Yes | Yes | Yes | No | No | No | US$191,893.44 (Igunza, Tanzania) US$58,554 (Homabay, Kenya) US$54,568 (Migori, Kenya) | No | Deliveries conducted by skilled birth attendant | NA | The average annual cost of the SCI per delivery with a skilled birth attendant was 15.0 US$ for Tanzania, and 10.6 US$ for Kenya. The cost per capita was 1.7 US$ for Tanzania, and 0.6 US$ for Kenya. |
| 11 | Kruk et al, 2007  Mozambique | Surgical technicians (Técnicos de cirurgia)  53 trained  Legally allowed in country to perform EmOC | Not defined | 2 years of classroom-based instruction at the Insti- tuto Superior de Ciencias de Saude and 1 year of internship under the supervision of a surgeon in a provincial hospital. | Classroom and facility | Full economic evaluation, CEA | Professional Salaries, materials and supplies, computers, travel, books, rural surgery test, library building | No | No | Yes | Yes | No | Yes | No | No | No | No | No | Yes | No | US$144,723 | No | Surgeries conducted |  | The resulting cost per surgery for te ́cnicos de cirurgia is $38.87 versus $144.1 for physicians, |
| 12 | Manasyan et al, 2011  Zambia | Midwives  123 trained  Participants working in first-level urban community public-sector clinics in Lusaka and Ndola. | 18 college-educated midwives were trained as Early Newborn Care instructors. | 5 days | Facility based | Full economic evaluation, CEA, CUA | Training of 18 ENC instructors, travel, board, and lodging costs for a volunteer trainer from abroad, training equipment and materials, and initial implementation and supervision. Per diem costs for training the clinic midwives were not included because they were trained during hours when they were working for the Zambian government. | Yes | No | Yes | Yes | Yes | Yes | Yes | Yes | Yes | No | Yes | No | No | US$20,223 | No | 97 lives saved | All-cause 7-day neonatal mortality decreased from 11.5 per 1000 to 6.8 per 1000 after ENC training (relative risk: 0.59; confidence interval: 0.48–0.77; P < .001) and was associated with a decrease in deaths caused by birth asphyxia (3.4–1.9 per 1000; P = .02) and infection (2.1–1.0 per 1000; P = .02) | The intervention costs were $208 per life saved and $5.24 per disability-adjusted life-year averted. |
| 13 | Crofts et al, 2015  Zimbabwe | Midwives and doctors  27 (12+15) trained | Seven staff from the United Kingdom (two midwives, three obstetricians, a paediatrician and an anaesthetist), three of whom were Zimbabwean | 1 | Facility based | Partial economic evaluation, Cost analysis | Trainer's manuals, course manuals, and a digital versatile disc containing annotated presentations, videos, course timetables, evaluation sheets and certificates, together with two mannequins. | Yes | No | Yes | Yes | No | -- | No | No | No | No | No | No | No | US$6000* | No | NA | NA | NA |
| 14 | Yau et al, 2016  United Kingdom | Doctors, midwives, operating department practitioners and maternity care assistants  477 (60 attending obstetricians, 21 anaesthetists, 286 midwives, 2 Operating department practitioners and 100 maternity care assistants (MCAs) | Local trainers at Southmead hospital where PROMPT was developed. | 1 | Facility based | Partial economic evaluation, Cost analysis | Start-up costs included purchasing training mannequins and teaching props, printing of training materials and assembly of emergency boxes (real and training). Variable costs included administration time, room hire, additional printing and the cost of releasing all maternity staff in the unit, either as attendees or trainers. | No | Yes | Yes | Yes | No | Yes | No | No | No | Yes | Yes | No | Yes | The total cost of establishing and running training at Southmead for 1 year was €148,806 | Yes | NA | NA | NA |
