# Supplementary material for: Economic evaluation of emergency obstetric care training: a systematic review
Source: BMC Pregnancy Childbirth. 2017 Dec 4;17:403. doi: 10.1186/s12884-017-1586-z (PMC5716021; doi:10.1186/s12884-017-1586-z)
Supplement: Supplementary file 3 — Quality assessment of cost analysis in partial and full economic evaluations (DOCX 15 kb) [file 12884_2017_1586_MOESM3_ESM.docx]

**Supplementary Table 3: Quality assessment of costs analysis presented in the partial and full economic evaluations**

| **Quality criteria** | **Full Economic Evaluations** | **Walker et al, 2002** | **Osei et al. 2005** | **Boulenger & Dmytraczenko 2007** | **Manasyan et al. 2011** | **Partial Economic Evaluation** | **Oyesola et al, 1997** | **Chukudebelu et al, 1997** | **Mekbib et al. 2003** | **Gill & Ahmed 2004** | **Islam et al. 2006** | **Santos et al. 2006** | **Rana et al. 2007** | **Kruk et al. 2007** | **Crofts et al. 2015** | **Yaw et al. 2016** |
| --- | --- | --- | --- | --- | --- | --- | --- | --- | --- | --- | --- | --- | --- | --- | --- | --- |
| Express costs in a currency value or as currency per person served by the program. |  | 1 | 1 | 1 | 1 |  | 1 | 1 | 1 | 1 | 1 | 1 | 1 | 1 | 1 | 1 |
| Provide detailed breakdown of costs incurred to develop and implement the intervention. |  | 1 | 1 | 1 | 1 |  | 0 | 0 | 1 | 0 | 0 | 0.5 | 1 | 1 | 0 | 1 |
| Include indirect costs, and intangible costs |  | 0 | 1 | 0 | 0 |  | 0 | 0 | 0 | 0 | 0 | 0 | 0 | 1 | 0 | 1 |
| Describe the perspective of the study and relate this to the costs being evaluated: Government, society, health care provider |  | 1 | 0 | 1 | 0 |  | 0 | 0 | 0 | 0 | 0 | 0 | 0 | 1 | 0 | 0 |
| Report the dates of the estimated resource quantities and unit costs. Describe methods for adjusting estimated unit costs to the year of reported costs if necessary. Describe methods for converting costs into a common currency base and the exchange rate. |  | 1 | 1 | 1 | 1 |  | 0 | 0 | 0 | 0 | 0 | 0 | 1 | 1 | 0 | 1 |
| Summarise key study findings and describe how they support the conclusions reached. Discuss limitations and the generalisability of the findings and how the findings fit with current knowledge. |  | 1 | 1 | 1 | 1 |  | 1 | 1 | 1 | 1 | 1 | 1 | 1 | 1 | 0.5 | 1 |
| Describe how the study was funded and the role of the funder in the identification, design, conduct, and reporting of the analysis. Describe other non-monetary sources of support. |  | 1 | 1 | 1 | 1 |  | 1 | 1 | 1 | 1 | 1 | 1 | 1 | 1 | 1 | 1 |
| Describe any potential for conflict of interest of study contributors in accordance with journal policy. In the absence of a journal policy, we recommend authors comply with International Committee of Medical Journal Editors recommendations. |  | 1 | 1 | 1 | 1 |  | 1 | 1 | 1 | 1 | 1 | 1 | 1 | 1 | 1 | 1 |
| **TOTAL QUALITY SCORE** |  | 7 | 7 | 7 | 6 |  | 4 | 4 | 5 | 4 | 4 | 4.5 | 6 | 8 | 3.5 | 7 |
| **TOTAL QUALITY PERCENTAGE** |  | 88% | 88% | 88% | 75% |  | 50% | 50% | 63% | 50% | 50% | 56% | 75% | 100% | 44% | 88% |

| *Green: High quality* |
| --- |
| *Yellow: Average quality* |
| *Red: Low quality* |
